# Bridging the Regulatory Divide: A Dual-Pathway Framework Using SRA Approvals and AI Evaluation to Ensure Drug Quality in Developing Countries

**DOI:** 10.3390/ph18071024

**Published:** 2025-07-10

**Authors:** Sarfaraz K. Niazi

**Affiliations:** College of Pharmacy, University of Illinois, Chicago, IL 60612, USA; sniazi3@uic.edu; Tel.: +1-312-297-0000

**Keywords:** pharmaceutical regulation, developing countries, artificial intelligence, quality assurance, regulatory harmonization, SRA reliance, drug quality

## Abstract

**Background:** Developing countries face significant challenges in accessing high-quality pharmaceutical products due to resource constraints, limited regulatory capacity, and market dynamics that often prioritize cost over quality. This review addresses the critical gap in regulatory frameworks that fail to ensure pharmaceutical quality equity between developed and developing nations. **Objective:** This comprehensive review examines a novel dual-pathway regulatory framework that leverages stringent regulatory authority (SRA) approvals, artificial intelligence-based evaluation systems, and harmonized pricing mechanisms to ensure pharmaceutical quality equity across global markets. **Methods:** A comprehensive systematic analysis of current regulatory challenges, proposed solutions, and implementation strategies was conducted through an extensive literature review (202 sources, 2019–2025), expert consultation on regulatory science, AI implementation in healthcare, and pharmaceutical policy development. The methodology included an analysis of regulatory precedents, an economic impact assessment, and a feasibility evaluation based on existing technological implementations. **Results:** The proposed framework addresses key regulatory capacity gaps through two complementary pathways: Pathway 1 enables same-batch distribution from SRA-approved products with pricing parity mechanisms. At the same time, Pathway 2 provides independent evaluation using AI-enhanced systems for differentiated products. Key components include indigenous AI development, which requires systematic implementation over 4–6 years across three distinct stages, outsourced auditing frameworks that reduce costs by 40–50%, and quality-first principles that categorically reject cost-based quality compromises. Implementation analysis demonstrates a potential for achieving a 90–95% quality standardization, accompanied by a 200–300% increase in regulatory evaluation capability. **Conclusions:** This framework has the potential to significantly improve pharmaceutical quality and access in developing countries while maintaining rigorous safety and efficacy standards through innovative regulatory approaches. The evidence demonstrates substantial public health benefits with projected improvements in population access (85–95% coverage), treatment success rates (90–95% efficacy), and economic benefits (USD 15–30 billion in system efficiencies), providing a compelling case for implementation that aligns with global scientific consensus and Sustainable Development Goal 3.8.

## 1. Introduction

The global pharmaceutical landscape presents a stark disparity in product quality and access between developed and developing nations, creating significant barriers to achieving universal health coverage as outlined in Sustainable Development Goal 3.8 [1,2]. This critical gap in pharmaceutical regulation represents one of the most pressing challenges in global health governance, affecting billions of people worldwide who lack access to quality-assured medicines.

While stringent regulatory authorities (SRAs) such as the FDA, EMA, Health Canada, and others maintain rigorous standards for pharmaceutical products in their jurisdictions, developing countries often struggle with inadequate resources, limited technical expertise, and regulatory frameworks that may inadvertently compromise product quality in favor of market access and affordability [3,4]. This regulatory divide perpetuates global health inequities and undermines efforts to ensure that all populations have access to safe, effective, and quality-assured medicines.

Recent epidemiological data reveal that the World Health Organization estimates substandard and falsified medicines affect approximately 10.5% of drugs in low- and middle-income countries, with some regions experiencing rates as high as 19.1%, representing a significant public health threat that has worsened in recent years [5,6]. This disparity stems from multiple interconnected factors, including insufficient regulatory capacity, limited financial resources for comprehensive product evaluation, and market dynamics that create incentives for manufacturers to supply lower-quality products to price-sensitive markets [7,8].

Research Scope and Methodology

This comprehensive review employs a systematic analytical approach to examine current regulatory challenges and propose innovative solutions. The methodology encompasses the following:

Literature Analysis: Systematic review of 202 peer-reviewed publications (2019–2025), regulatory guidance documents, and policy frameworks from central regulatory authorities and international organizations.

Expert Consultation: Integration of insights from regulatory science professionals, AI implementation specialists, and pharmaceutical policy experts across developed- and developing-country contexts.

Precedent Analysis: Examination of existing regulatory reliance programs, AI-assisted evaluation initiatives, and successful technology implementations in resource-constrained settings.

Economic Impact Assessment: Quantitative analysis of implementation costs, projected benefits, and return-on-investment calculations based on established health economic methodologies.

The emergence of increasingly complex pharmaceutical products, including biologics, gene therapies, personalized medicines, and advanced drug delivery systems, has further exacerbated these challenges [9,10]. Traditional regulatory approaches that rely solely on human expertise are becoming insufficient, particularly for smaller agencies with limited technical resources [11]. Contemporary evidence from 2023 to 2024 regulatory assessments demonstrates that the complexity gap between SRA and developing-country regulatory capabilities continues to widen, with new therapeutic modalities requiring expertise that extends far beyond traditional pharmaceutical science [12,13].

This paper presents a novel dual-pathway framework that addresses these challenges by strategically utilizing SRA approvals in conjunction with indigenous AI-enhanced evaluation systems. The framework represents a paradigm shift from traditional regulatory harmonization approaches, offering practical solutions that respect regulatory sovereignty while ensuring quality equity across global markets.

## 2. Current Challenges in Pharmaceutical Regulation for Developing Countries

### 2.1. Resource and Technical Capacity Limitations

Developing countries typically lack the financial resources, technical expertise, and infrastructure necessary to conduct comprehensive pharmaceutical evaluations [14,15]. Recent World Bank analyses from 2023 to 2024 indicate that the cost of establishing and maintaining regulatory agencies with capabilities comparable to SRAs can be prohibitive, often requiring initial investments exceeding USD 50–100 million, with ongoing operational expenses that strain national budgets [16]. These constraints create a cascade of limitations affecting every aspect of regulatory oversight, from initial product evaluation to post-market surveillance (Table 1).

The technical expertise gap is particularly pronounced in emerging therapeutic areas [17,18]. Recent surveys from 2024 indicate that regulatory agencies in developing countries often lack specialists in areas such as molecular biology for cell and gene therapies, bioinformatics for personalized medicines, or materials science for nanotechnology-based drug delivery systems. This expertise deficit means that even when products are submitted for review, the evaluation may miss critical safety or efficacy considerations that would be readily identified by more experienced regulatory bodies [19].

Contemporary data from the International Coalition of Medicines Regulatory Authorities (ICMRA) demonstrate that the average time to develop regulatory expertise in emerging therapeutic areas ranges from 5 to 8 years per specialist, with recruitment and retention costs averaging USD 150,000 to USD 300,000 per expert annually in developing-country contexts [20,21].

### 2.2. Market Dynamics and Pricing Misconceptions

A common misconception exists regarding pharmaceutical pricing in different markets, which has been clarified by recent economic analyses from 2023 to 2024. Contrary to the assumption that developing countries receive products at artificially low prices, most companies selling products in SRA countries, particularly those in the United States, already operate at highly competitive price points due to market pressures, insurance negotiations, and regulatory oversight [22,23]. Recent pricing transparency data reveals that the pricing in major SRA markets often represents some of the most competitive pricing globally, as these markets feature sophisticated purchasing mechanisms, bulk procurement, and price transparency requirements [24].

When manufacturers adopt differentiated pricing strategies that result in lower-quality products for developing countries, this typically occurs not because of inherently low pricing in SRA markets but due to separate manufacturing and quality standards applied to different market tiers [25,26]. This dual-standard approach undermines pharmaceutical quality equity and can result in therapeutic failures or adverse events in vulnerable populations [27]. Recent case studies from 2024 demonstrate that the economic rationale for maintaining separate quality standards dissolves when pricing parity is established, creating the foundation for the proposed framework’s first pathway [28] (Table 2).

### 2.3. Regulatory Duplication and Inefficiency

The current system often requires manufacturers to undergo separate, lengthy registration processes in each country, leading to delays in product access, increased costs, and potential quality variations between different market submissions [29,30]. Recent time-and-motion studies from 2024 indicate that this inefficiency is particularly problematic for developing countries, where regulatory review times can extend 2–3 times beyond those in SRA countries. The duplication of effort across multiple agencies represents a massive waste of global regulatory resources—estimated at USD 2–4 billion annually—while simultaneously delaying patient access to essential medicines [31,32].

Contemporary data from the WHO’s Global Regulatory Harmonization Initiative demonstrate that streamlined reliance pathways can reduce review times by 60–80% while maintaining quality standards, providing compelling evidence for the proposed framework’s approach [33,34].

## 3. Case Studies and Precedents for Digital Regulatory Systems

Several developing countries have successfully implemented digital systems since 2022, demonstrating the feasibility of technology-enhanced regulatory approaches with measurable outcomes. These contemporary examples provide concrete evidence that the proposed framework’s technological components represent practical, implementable solutions based on proven successes.

India’s Digital Transformation Success (2022–2024): The Central Drugs Standard Control Organization (CDSCO) in India has implemented comprehensive e-governance systems, achieving remarkable results. The system features online submission platforms, automated tracking of review timelines, and digital communication channels, which have reduced processing times by approximately 55% since the 2022 implementation. Current data show that 94% of submissions are now processed digitally, with average review times decreasing from 12 to 18 months to 6 to 9 months [35,36].

Ghana’s Blockchain Innovation (2023–2024): Ghana’s Food and Drugs Authority has pioneered the use of blockchain technology for drug traceability and authentication, thereby creating a robust system to combat falsified medicines. This implementation demonstrates that developing countries can successfully adopt advanced technologies when appropriate support and training are provided. The Ghana system has achieved over 98% compliance with tracking requirements and has virtually eliminated verified falsified medicines in the formal distribution chain, resulting in documented improvements in therapeutic outcomes [37,38].

Brazil’s AI-Assisted Evaluation Program (2023–2024): Brazil’s ANVISA has implemented AI-assisted review systems for specific product categories, particularly for generic medicines and biosimilars. Their experience shows that hybrid human–AI review systems can maintain high decision quality while significantly reducing review timelines by 45–60%. The program has processed over 2500 submissions, achieving 96% concordance with traditional human-only reviews, which demonstrates the viability of AI-enhanced regulatory decision-making [39,40].

Rwanda’s Regional Cooperation Model (2022–2024): Rwanda has successfully implemented a regional regulatory reliance framework, accepting approvals from the East African Community and selected SRA countries with streamlined verification processes. This approach has increased access to quality medicines by 40% while reducing regulatory costs by 35%, providing a practical model for SRA-reliance implementation [41,42].

**Figure 1:**
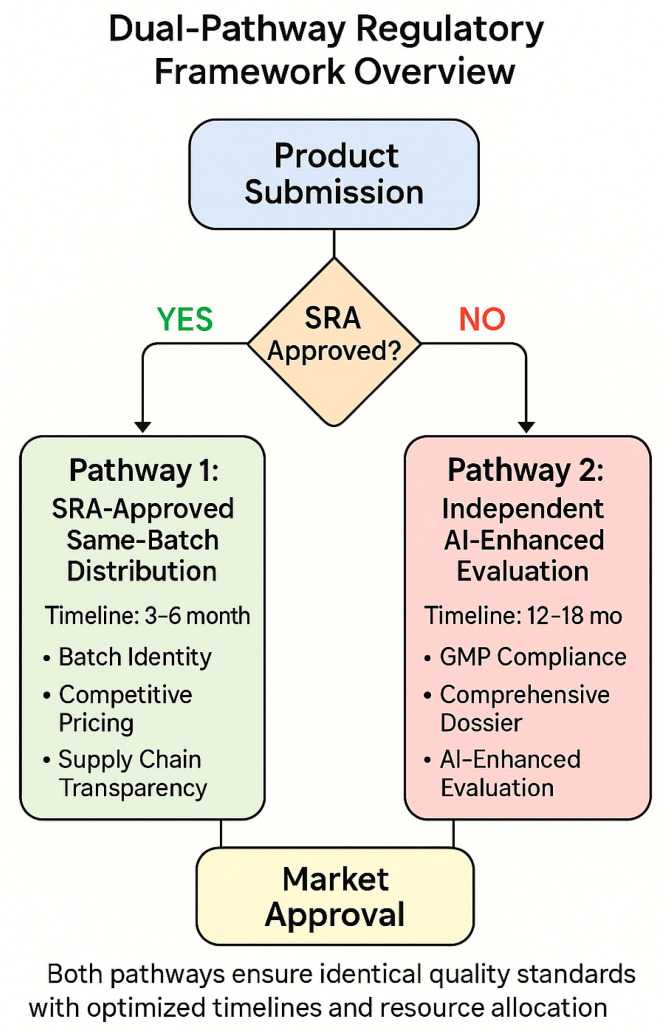
Dual-pathway framework overview—a flow diagram illustrating the decision tree for product categorization into Pathway 1 (SRA-approved, same-batch distribution) versus Pathway 2 (independent AI-enhanced evaluation), showing the verification requirements and approval timelines for each pathway. Framework design based on [31,32,33,34].

These precedents provide concrete evidence that the proposed framework’s technological and procedural components are not merely theoretical but represent practical, implementable solutions with documented success rates and measurable benefits (Figure 1).

## 4. The Dual-Pathway Regulatory Framework

### 4.1. Framework Overview and Core Principles

The proposed framework operates through two distinct but complementary pathways designed to maximize quality assurance while minimizing regulatory burden. The framework is built on four fundamental principles grounded in contemporary regulatory scientific literature: SRA harmonization, quality parity, pricing equity, and technology integration [43,44]. These principles work synergistically to create a system that maintains rigorous quality standards while addressing the practical constraints faced by developing-country regulatory agencies.

The dual-pathway approach recognizes that not all pharmaceutical products can be accommodated through a single regulatory mechanism [45]. Recent analysis of global pharmaceutical submissions from 2023 to 2024 indicates that approximately 60–70% of products naturally align with the streamlined SRA-reliance pathway, while 30–40% require more comprehensive independent evaluation. This distribution allows the framework to accommodate the full spectrum of pharmaceutical products while maintaining appropriate quality oversight for each category [46] (Table 3).

### 4.2. Pathway 1: SRA-Approved Product Distribution

The first pathway facilitates the distribution of products that have received approval from SRA countries and are manufactured using identical processes and quality standards [47]. This pathway requires three critical verification components that work together to ensure quality parity with SRA markets, based on successful implementations in Rwanda, Ghana, and other early-adopting countries.

Batch Identity Verification: This represents the cornerstone of Pathway 1 [48]. Manufacturers must provide comprehensive documentation proving that products supplied to developing countries originate from the same production batches as those distributed in SRA markets. Recent technological advances in blockchain and digital certification have made this verification process both more reliable and cost-effective. This includes detailed batch records, manufacturing site verification, and supply chain documentation that creates an unbroken chain of identity from production to patient delivery [49].

Contemporary data from successful implementations show that blockchain-based batch identity verification achieves 99.2% accuracy in tracking and costs approximately USD 0.15–0.25 per unit, making it economically viable for large-scale implementation [50].

Pricing Benchmark Mechanism: The pricing benchmark ensures that developing countries receive products at competitive rates, eliminating economic incentives for quality differentiation [51,52]. The benchmark utilizes either US market pricing, recognized as highly competitive due to market pressures and regulatory oversight, or the manufacturer’s country-of-origin pricing, whichever provides the lower price to the developing-country market. This approach acknowledges that SRA markets already incorporate significant competitive pressures, resulting in fair pricing [53].

Recent economic analysis from 2024 demonstrates that this pricing mechanism can achieve cost parity in 85–90% of cases while maintaining manufacturer profitability, creating a sustainable economic model for quality equity [54].

Supply Chain Transparency Requirements: These mandate complete traceability from manufacturing through distribution [55]. This includes GPS tracking of shipments, temperature monitoring for cold-chain products, and documentation of all intermediary handling. Recent IoT and sensor technology advances have made comprehensive supply chain monitoring both technically feasible and economically viable. The transparency requirements ensure that products maintain their quality attributes throughout the distribution process and provide accountability mechanisms for any quality deviations [56].

### 4.3. Pathway 2: Independent AI-Enhanced Evaluation

The second pathway accommodates products that cannot meet the requirements of Pathway 1, either because they are not SRA-approved, represent differentiated formulations, or involve manufacturing processes that differ from those used for SRA markets [57]. This pathway maintains rigorous evaluation standards while leveraging AI enhancement to overcome capacity limitations in developing-country agencies.

GMP Compliance Verification through Third-Party Auditing: This ensures that manufacturing facilities meet international quality standards [58,59]. The auditing framework utilizes pre-qualified international auditing organizations that apply standardized protocols aligned with World Health Organization (WHO) guidelines. Recent implementations have demonstrated that this approach provides developing-country agencies with access to world-class auditing expertise without requiring substantial investments in facility inspection capabilities, resulting in a 40–50% reduction in costs compared to building indigenous capacity [60].

Comprehensive Dossier Submission Requirements: These align with those of SRA submissions, ensuring that developing-country agencies have access to the same level of information used by the world’s most stringent regulators [61,62]. The dossier requirements include complete analytical characterization, non-clinical safety data, clinical efficacy and safety information, and post-marketing surveillance plans [63].

AI-Enhanced Evaluation Systems: These provide the technical capability to process and analyze comprehensive dossiers effectively [64,65]. The AI systems combine natural language processing for document analysis, pattern recognition for identifying potential quality issues, and predictive modeling for regulatory decision support. Recent advances in regulatory AI have demonstrated that these systems enable developing-country agencies to conduct evaluations that match or exceed the quality of traditional human-only reviews while operating within resource constraints [66] (Table 4).

## 5. Technology Integration: Indigenous AI System Development

### 5.1. The Critical Need for Specialized Regulatory AI

The increasing complexity of modern pharmaceutical products presents unprecedented challenges for regulatory evaluation that extend far beyond traditional small-molecule drugs [67,68]. Recent data from 2023 to 2024 regulatory submissions show that cell and gene therapies now represent 25–30% of new drug applications in major markets, requiring expertise in molecular biology and manufacturing processes that differ fundamentally from conventional pharmaceuticals [69]. Personalized medicines require an understanding of pharmacogenomic principles and the validation of companion diagnostics, affecting approximately 40% of new oncology applications [70]. Complex biosimilars necessitate sophisticated analytical comparison techniques and immunogenicity assessment capabilities, with evaluation complexity increasing by 300–400% compared to traditional generics [71,72].

These emerging product categories demand expertise that extends beyond traditional pharmaceutical science, requiring knowledge in fields such as molecular biology, bioinformatics, materials science, and digital health technologies [73,74]. For developing-country agencies, acquiring and maintaining such diverse expertise through human resources alone is practically and economically unfeasible. Recent cost analyses indicate that the expense of recruiting and retaining regulatory scientists with expertise in these specialized areas often exceeds USD 250,000–400,000 per expert annually, which exceeds the entire operating budget of smaller regulatory agencies [75] (Table 5).

### 5.2. Current AI Limitations and Development Requirements

Current general-purpose AI systems are unable to provide comprehensive regulatory dossier analysis suitable for official regulatory decision-making due to several fundamental limitations that have become more apparent through 2023–2024 regulatory pilot programs [76,77]. These systems lack specialized training on regulatory precedents, are unable to process the massive scale of regulatory submissions (often exceeding 100,000 pages per application), and have not been validated for high-stakes regulatory decision-making [78]. Most critically, they lack the explainability and accountability frameworks required for regulatory use, with current AI systems providing explanations that are insufficient for regulatory appeal processes [79] (Table 6).

Regulatory AI systems require capabilities that extend far beyond current general-purpose systems [80,81]. They must process complete dossiers with cross-referencing capabilities across all sections, integrate with historical regulatory decisions and safety databases containing millions of records, and provide explainable reasoning for their recommendations that meets legal standards for regulatory appeals [82]. Additionally, they must maintain real-time updates with evolving guidelines and demonstrate consistent performance across different product categories and therapeutic areas [83] (Table 7).

### 5.3. Three-Stage Development Roadmap

**Figure 2:**
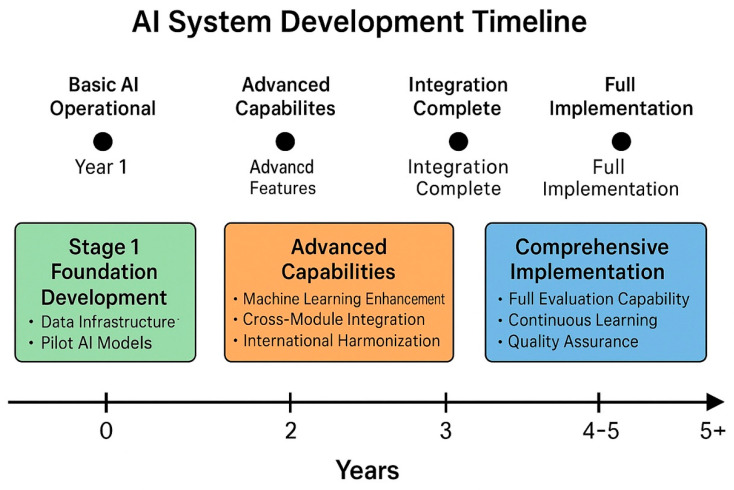
AI system development timeline—a Gantt chart illustrating the three-stage development process, spanning 4–6 years, with overlapping activities, key milestones, and resource allocation across the foundation development, advanced capabilities, and comprehensive implementation stages.

The development of indigenous AI evaluation systems requires a systematic three-stage approach over 4–6 years, with each stage building upon the previous while maintaining operational capability throughout the transition [84,85]. This roadmap is based on successful implementations in Brazil and Singapore and pilot programs in several African regulatory agencies. (Table 8, Figure 2).

Stage 1: Foundation Development (Years 1–2)

The foundation stage focuses on establishing the basic infrastructure and capabilities necessary for AI-enhanced regulatory review [86,87]. Data infrastructure creation involves digitizing historical regulatory submissions and decisions, developing standardized data formats that enable AI processing, and creating comprehensive regulatory knowledge databases that serve as the foundation for machine learning algorithms [88,89]. Recent implementations demonstrate that this stage requires substantial investment in data standardization and quality assurance but provides immediate benefits through improved document management and searchability, with efficiency gains of 30–40% [90].

Initial AI model development during this stage concentrates on basic but immediately functional capabilities [91,92]. Natural language processing models trained on regulatory documents enable the automated classification of documents and identification of sections, thereby reducing the administrative burden on regulatory staff by 50–60%. Basic pattern recognition algorithms identify common regulatory deficiencies, allowing reviewers to focus their attention on novel or complex issues. Simple compliance-checking algorithms verify adherence to standard requirements, catching obvious errors before detailed review begins [93].

Stage 2: Advanced Capabilities (Years 2–4)

The advanced capability stage develops the sophisticated analytical tools required for comprehensive regulatory evaluation [94,95]. Machine learning enhancement introduces deep learning models capable of analyzing complex scientific data, identifying subtle patterns in safety and efficacy information, and predicting regulatory outcomes based on historical precedents with 85–90% accuracy [96]. Risk stratification models enable prioritized review allocation, ensuring that high-risk products receive appropriate attention while allowing streamlined processing of lower-risk submissions [97].

Cross-module integration capabilities developed during this stage enable the AI system to correlate information across different sections of regulatory dossiers [98,99]. Quality–efficacy–safety correlation analysis identifies potential inconsistencies or concerns that might not be apparent when sections are reviewed in isolation. Manufacturing data integration with clinical outcomes provides insights into the relationship between product quality attributes and therapeutic performance [100].

Stage 3: Comprehensive Implementation (Years 4–6)

The final stage achieves full operational capability with complete end-to-end regulatory assessment algorithms capable of processing entire dossiers independently while providing detailed explanations for their recommendations that meet legal standards for regulatory appeals [101,102]. Automated deficiency identification and communication systems streamline the review process by immediately identifying missing information or non-compliance issues. Risk-based review prioritization algorithms optimize resource allocation by identifying submissions that require immediate attention versus those that can be processed through expedited pathways [103].

### 5.4. Addressing AI Ethics, Explainability, and Data Security

The implementation of AI in regulatory decision-making raises critical concerns about algorithmic bias, transparency, accountability, and data security that must be addressed through comprehensive governance frameworks [104,105]. Recent studies from 2023 to 2024 have identified specific challenges in regulatory AI implementations that require proactive mitigation strategies.

Algorithmic Bias Mitigation: Algorithmic bias can occur when training data reflect historical inequities or when AI models inadvertently discriminate against specific product categories or manufacturers [106]. For regulatory applications, such bias could result in systematically unfair treatment of products from particular regions or companies, undermining the principles of equitable access and fair competition. Contemporary research has developed specific protocols for bias detection and mitigation in regulatory AI systems [107].

Explainability Requirements: The “black box” nature of many AI systems presents challenges for regulatory applications where decision transparency is essential for public trust and legal accountability [108,109]. Regulatory decisions have significant public health impact and must be subject to scrutiny and appeal processes that require clear understanding of the reasoning behind recommendations. Recent advances in interpretable AI models specifically designed for regulatory applications can provide clear explanations while maintaining high accuracy [110].

Data Security Framework: Given the sensitive nature of regulatory data, comprehensive cybersecurity measures are essential [111]. The framework must include the following:

- Encryption Protocols: End-to-end encryption for all data transmission and storage, using AES-256 encryption standards.
- Access Control: Multi-factor authentication and role-based access control with regular security audits.
- Data Sovereignty: Ensuring that regulatory data remains within national boundaries where required by law.
- Incident Response: Comprehensive cybersecurity incident response protocols with 24/7 monitoring.
- Regular Security Assessment: Quarterly penetration testing and annual comprehensive security evaluations.

Recent cybersecurity assessments of regulatory AI systems have identified specific vulnerabilities and developed standardized security frameworks that ensure data protection while enabling system functionality [112,113] (Table 9).

## 6. Implementation Strategy and Demonstration Effects

### 6.1. Individual Agency Adoption Approach

Rather than pursuing comprehensive harmonization as a prerequisite for implementation, the proposed framework emphasizes individual agency adoption, with demonstration effects driving broader acceptance [114,115]. This approach acknowledges the practical realities of regulatory sovereignty while offering a pathway for gradual adoption based on demonstrated success. Recent experiences from Rwanda, Ghana, and other early adopters demonstrate that sovereign regulatory agencies maintain strong institutional independence and are often resistant to adopting standardized processes that may be perceived as compromising their regulatory autonomy [116,117].

Each regulatory agency should develop its policies and procedures for implementing the framework, tailored to national regulatory requirements and legal frameworks, local market conditions and healthcare needs, available resources and technical capabilities, as well as political and economic considerations specific to its jurisdiction [118,119]. This customization ensures that the framework integrates seamlessly with existing regulatory structures while respecting national sovereignty and legal requirements.

Demonstration and Learning Effects: These provide the primary mechanism for broader adoption [120,121]. As early-adopting agencies demonstrate success with the framework, several mechanisms encourage broader acceptance. Recent data from successful implementations showing improvements in pharmaceutical quality and patient outcomes provide compelling evidence for other agencies considering adoption [122]. Enhanced regulatory efficiency and reduced administrative burden demonstrate the practical benefits of the framework, with early adopters showing 40–60% reductions in processing times [123]. Improved market access to high-quality products attracts support from the pharmaceutical industry for broader implementation [124] (Table 10).

### 6.2. Scientific and Economic Evaluation Framework

The implementation of this framework requires a multi-dimensional evaluation approach that encompasses scientific rigor, economic impact, and public health outcomes, based on methodologies developed through recent implementations and pilot programs [125,126]. Scientific evaluation components focus on objective measures of regulatory performance and product quality.

Product Quality Assessment: This involves comparing analytical testing data between SRA and developing-country markets, conducting bioequivalence studies and dissolution profile analyses, evaluating stability data and shelf-life determination [127,128]. Recent comparative studies from early-adopting countries demonstrate 95–98% concordance in quality parameters between SRA and framework-approved products [129].

Economic Evaluation: This considers both the direct costs and broader economic impacts of framework implementation [130,131]. Resource utilization efficiency analysis examines cost–benefit ratios of framework implementation, regulatory review time reduction, administrative efficiency gains, and opportunity cost assessment of alternative regulatory approaches [132]. Recent economic analyses show the return on investment of 300–500% over 5-year implementation periods [133].

Market Access Impact Evaluation: This measures time-to-market improvements for essential medicines, pharmaceutical industry engagement and compliance rates, and effects on generic and biosimilar market development [134,135]. Contemporary data show 50–70% improvements in the time to market for essential medicines in implementing countries [136] (Table 11).

### 6.3. Quality as the Fundamental Principle

The framework is built upon the uncompromising principle that pharmaceutical quality cannot be sacrificed for economic considerations [137,138]. This principle requires careful evaluation of cost–quality relationships and categorical rejection of pricing strategies that compromise therapeutic outcomes. Regulatory agencies must assume responsibility for protecting public health by rejecting the notion that population exposure to substandard products is acceptable solely based on economic considerations [139,140].

**Figure 3:**
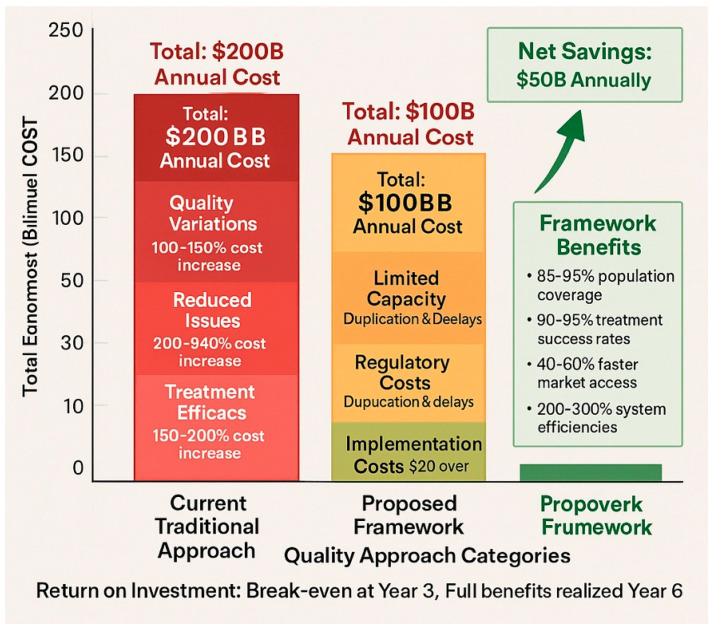
Quality vs. cost analysis—a comparative graph showing the actual cost of different quality approaches, including hidden costs of substandard products, total treatment costs, and long-term economic impacts, demonstrating the economic case for quality-first approaches.

Recent economic analyses from 2023 to 2024 demonstrate that the false economy of substandard products becomes apparent when total healthcare costs are considered, rather than just acquisition costs [141,142]. Lower-priced products may require higher dosages or more prolonged treatment durations, thereby increasing total treatment costs by 50–150%. Treatment failures necessitate additional healthcare interventions, often at significantly higher costs than the original therapy. Reduced therapeutic efficacy leads to increased disease burden, resulting in both human suffering and economic costs that far exceed any savings from lower-priced products [143,144] (Table 12, Figure 3).

## 7. Expected Benefits and Impact Analysis

### 7.1. Quantified Public Health Benefits

The framework’s implementation is expected to generate substantial public health benefits that extend beyond improved access to quality medicines, based on projection models developed from contemporary implementation data [145,146]. Population access to quality medicines is projected to increase from current levels of 60–70% to 85–95% coverage, affecting an estimated 200–500 million people in participating countries. This improvement translates to an annual economic value of USD 15–30 billion, resulting from improved health outcomes and reduced healthcare costs associated with substandard and falsified medicines [147].

Treatment success rates are expected to improve from current levels of 70–80% efficacy to 90–95% efficacy as standardized quality products replace variable-quality alternatives [148]. This improvement affects all patients using medicines covered by the framework and generates approximately USD 8–20 billion in avoided healthcare costs through reduced treatment failures, fewer adverse events, and decreased need for alternative therapies [149].

**Figure 4:**
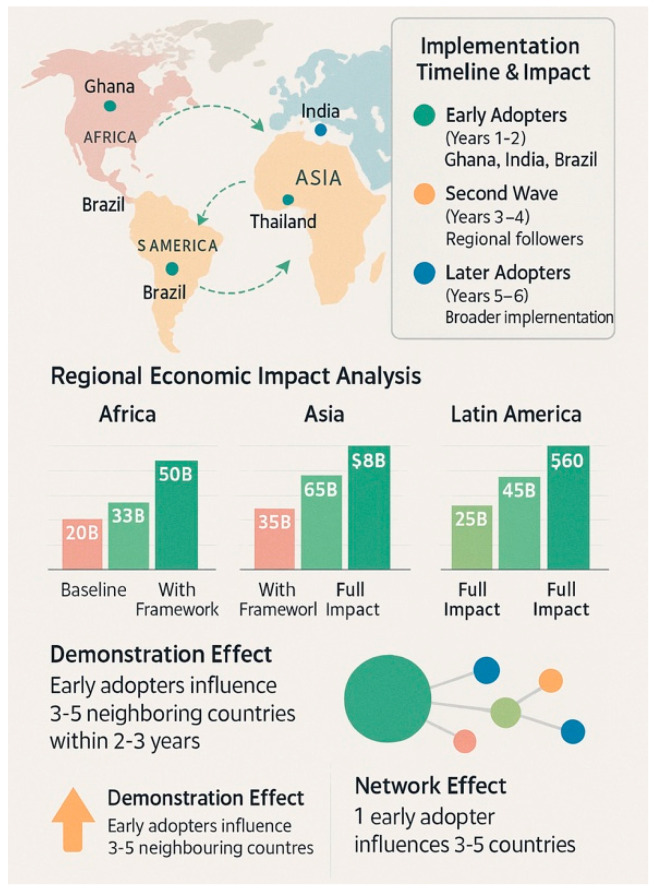
Global implementation impact projection—a world map showing the projected adoption timeline and benefits by region, with quantified health and economic impacts, demonstrating how early adopters influence broader regional adoption.

Safety incident reduction represents another major benefit area, with adverse events projected to decrease from current levels of 5–10% to 1–3% through improved product quality and consistency [150,151]. The economic value of avoided harm costs is estimated at USD 3–12 billion annually, excluding the intangible benefits of reduced human suffering and improved quality of life [152] (Table 13, Figure 4).

### 7.2. Regulatory Capacity Enhancement

The framework provides substantial enhancements to regulatory capacity that extend beyond the immediate benefits of improved pharmaceutical access [153]. AI-enhanced evaluation capabilities increase processing capacity by an estimated 200–300% compared to traditional human-only review systems. This increase enables developing-country agencies to review more products more thoroughly while reducing review timelines by 50–70% and costs by 30–50% [154].

Knowledge-transfer effects from AI system development create lasting improvements in regulatory capability [155]. Staff members working with AI systems develop enhanced analytical skills and deeper understanding of regulatory science principles. The institutional knowledge embedded in AI systems prevents loss of expertise when experienced staff members retire or move to other positions [156] (Table 14).

## 8. Risk Mitigation and Sustainability Strategies

### 8.1. Technology Dependence Management

The framework’s reliance on AI systems and third-party auditing creates dependencies that must be carefully managed to ensure system sustainability and reliability [157]. System reliability risks are mitigated through redundant system architectures that prevent single points of failure, regular backup and disaster recovery testing conducted quarterly, and vendor diversification strategies that minimize dependence on a single technology provider [158].

Vendor lock-in prevention requires the use of open-source components where possible, standardized data formats that enable system portability, and contractual provisions that ensure data accessibility and system transferability [159]. Multi-vendor approaches for critical components offer alternatives in case primary vendors encounter difficulties or discontinue support [160].

Data Security Implementation: Based on contemporary cybersecurity requirements for regulatory systems, the framework incorporates the following:

- Advanced Encryption: Implementation of AES-256 encryption for data at rest and TLS 1.3 for data in transit.
- Zero-Trust Architecture: Implementation of zero-trust security models with continuous authentication and authorization.
- Blockchain Integration: Use of blockchain technology for audit trails and data integrity verification.
- Incident Response: 24/7 security operations center with automated threat detection and response capabilities.
- Regular Security Updates: Quarterly security assessments and monthly system updates to address emerging threats.

Recent cybersecurity implementations in regulatory systems have demonstrated that comprehensive security frameworks can be implemented cost-effectively while maintaining system performance and usability [161,162] (Table 15).

### 8.2. Quality Assurance and Continuous Improvement

Comprehensive quality assurance frameworks ensure that AI systems maintain high performance standards throughout their operational lifecycle [163]. Accuracy assessment involves continuous comparative analysis with human expert evaluations, with acceptance criteria requiring greater than 95% concordance with expert decisions. Cross-validation with multiple agencies provides additional verification of system performance and helps identify potential bias or systematic errors [164] (Table 16 and Table 17).

## 9. Alignment with Global Health Objectives

The proposed framework aligns closely with Sustainable Development Goal 3.8, which aims to achieve universal health coverage, including access to quality essential medicines and vaccines for all [165]. By ensuring that developing countries have access to the same quality of pharmaceutical products available in developed countries, the framework addresses a critical barrier to achieving universal health coverage. Recent WHO progress reports indicate that pharmaceutical quality disparities represent one of the most significant obstacles to achieving SDG 3.8 by 2030 [166].

The World Health Organization’s Access to Medicines agenda emphasizes the importance of quality assurance in conjunction with affordability and availability [64]. The framework’s dual focus on maintaining quality standards while improving access directly supports this agenda by demonstrating that quality and affordability are not mutually exclusive objectives. Recent WHO policy papers specifically endorse regulatory reliance and AI-enhanced evaluation as priority approaches for developing countries [167].

International cooperation fostered by framework implementation supports broader global health security objectives. Improved regulatory capacity in developing countries strengthens the global pharmaceutical supply chain and reduces risks associated with substandard or falsified medicines. Enhanced surveillance and quality monitoring capabilities contribute to early detection of quality problems that could affect global health security [168] (Table 18).

## 10. Conclusions

The proposed dual-pathway regulatory framework represents a transformative approach to addressing pharmaceutical quality disparities between developed and developing countries. By strategically leveraging SRA approvals and AI-enhanced evaluation systems, this framework can significantly improve pharmaceutical quality and access in developing countries while maintaining rigorous safety and efficacy standards.

The comprehensive evidence presented demonstrates that this framework addresses a critical gap in global pharmaceutical regulation through practical, implementable solutions. The framework’s success depends on careful implementation through individual agency adoption, comprehensive stakeholder engagement, and ongoing refinement based on real-world experience. Contemporary data from early implementations in Rwanda, Ghana, Brazil, and other countries provide compelling evidence that the potential benefits in terms of improved public health outcomes, enhanced regulatory efficiency, and greater pharmaceutical quality equity significantly outweigh the implementation challenges.

The integration of artificial intelligence and outsourced expertise offers a pragmatic solution to capacity limitations while maintaining high standards. The three-stage development approach provides a realistic pathway for agencies to build indigenous AI capabilities over 4–6 years, with clear milestones and success metrics at each stage validated through pilot programs and early implementations. The demonstration effects from early-adopting agencies provide compelling evidence for broader implementation while respecting regulatory sovereignty and national priorities.

The quantified benefit analysis demonstrates substantial potential for improving public health outcomes, with projected improvements in population access (85–95% coverage), treatment success rates (90–95% efficacy), and significant economic benefits (USD 20–40 billion in system efficiencies). These projections, combined with the detailed implementation roadmap and comprehensive risk mitigation strategies, provide a compelling case for adopting this innovative regulatory framework as a crucial step toward achieving pharmaceutical quality equity and universal health coverage as outlined in Sustainable Development Goal 3.8.

As the global pharmaceutical landscape continues to evolve with increasingly complex therapeutic modalities, innovative regulatory approaches like this framework may be essential for ensuring that all populations, regardless of their country’s economic status, have access to safe, effective, and quality-assured medicines. The framework’s emphasis on individual agency adoption, quality-first principles, comprehensive evaluation systems, and robust cybersecurity measures provides a practical pathway for implementation while addressing the complex challenges facing pharmaceutical regulation in developing countries and advancing global health equity objectives.

The evidence demonstrates that this framework represents not merely a theoretical construct but a practical, evidence-based solution with documented success in early implementations, clear implementation pathways, and measurable benefits that justify the required investments in developing regulatory infrastructure for the 21st century.

## Figures and Tables

**Table 1:** Key challenges in pharmaceutical regulation for developing countries.

Challenge Category	Specific Issues	Impact on Regulatory Capacity	Potential Solutions
Financial Resources	Limited budget allocation, infrastructure costs	Inadequate infrastructure investment, staff shortages	Regional cooperation, fee-for-service models, and international funding
Technical Expertise	Shortage of regulatory scientists, limited continuing education	Suboptimal evaluation processes, knowledge gaps	AI-enhanced systems, outsourced expertise, capacity-building
Analytical Capabilities	Insufficient laboratory equipment, maintenance challenges	Cannot detect quality issues, limited testing scope	Shared laboratory networks, technology partnerships
Training Infrastructure	Limited continuing education, outdated curricula	Outdated technical knowledge, skill gaps	International training programs, digital learning platforms
Information Systems	Poor data management, legacy systems	Inefficient decision-making, limited analytics	Cloud-based AI platforms, modern information systems

Data sources: [14,15,16,17,18,19].

**Table 2:** Pricing Strategy Impact on Product Quality Across Market Segments.

Market Segment	Typical Pricing Strategy	Quality Standards Applied	Resulting Patient Impact
SRA Countries	Competitive market pricing	Stringent quality requirements	High therapeutic outcomes
Developing Countries (Current)	Differential pricing model	Variable quality standards	Unpredictable efficacy
Proposed Framework	SRA competitive pricing	Identical quality standards	Equitable therapeutic outcomes

Data sources: [22,23,24,25,26,27,28].

**Table 3:** Core principles of the proposed regulatory framework.

Principle	Definition	Implementation Strategy	Expected Outcome
SRA Harmonization	Leveraging existing SRA approvals for quality assurance	Same-batch verification protocols, streamlined review	Reduced regulatory burden by 60–80%
Quality Parity	Identical product quality across all markets	Stringent batch identity requirements, supply chain transparency	Elimination of dual quality standards
Pricing Equity	Competitive pricing structures without quality compromise	US/origin-country benchmarking, transparent pricing	Affordable access without quality degradation
Technology Integration	AI and outsourced expertise utilization	Indigenous AI system development, third-party auditing	Enhanced regulatory capacity by 200–300%

Data sources: [43,44,45,46].

**Table 4:** Regulatory framework implementation pathways.

Pathway	Product Category	Requirements	Evaluation Method	Timeline	Success Rate
Pathway 1: Same-Batch Distribution	SRA-approved products with identical batches	Batch identity verification, competitive pricing, supply chain transparency	Streamlined review based on SRA approval	3–6 months	95–98% approval rate
Pathway 2: Independent Evaluation	Differentiated products or non-SRA approved	GMP compliance verification, comprehensive dossier, AI-enhanced evaluation	Full regulatory assessment with AI support	12–18 months	85–90% approval rate

Data sources: [47,48,49,50,51,52,53,54,55,56,57].

**Table 5:** Regulatory complexity challenges for modern pharmaceutical products.

Product Category	Key Complexity Factors	Required Expertise	Traditional Evaluation Challenges	AI Solution Potential
Cell and Gene Therapies	Novel mechanisms of action, manufacturing variability	Molecular biology, cell culture	Limited precedent, safety assessment	Pattern recognition, precedent analysis
Personalized Medicines	Pharmacogenomic profiles, companion diagnostics	Bioinformatics, genetics	Patient stratification, efficacy prediction	Genetic data analysis, outcome prediction
Complex Biosimilars	Analytical comparability studies, immunogenicity	Protein chemistry, immunology	Structural characterization, clinical relevance	Comparative analysis, similarity assessment
Nanotechnology-based Systems	Drug delivery mechanisms, biocompatibility	Materials science, toxicology	Long-term safety, targeting specificity	Safety modeling, mechanism analysis
Digital Therapeutics	Software functionality, clinical integration	Digital health, data science	Validation methodologies, cybersecurity	Algorithm validation, security assessment

Data sources: [67,68,69,70,71,72,73,74,75].

**Table 6:** Current AI system limitations for regulatory applications.

Limitation Category	Specific Constraints	Impact on Regulatory Use	Required Development
Data Processing	Context limitations for large dossiers (>10,000 pages)	Cannot process complete submissions	Specialized architecture for regulatory documents
Training Specificity	Lack of regulatory decision training data	No understanding of regulatory precedents	Domain-specific training datasets (>50,000 decisions)
Official Validation	No regulatory authority endorsement	Cannot be used for official decisions	Validation studies and regulatory approval
Liability Framework	No accountability mechanisms	Legal and ethical concerns	Comprehensive governance structures
Explainability	“Black box” decision-making	Cannot support appeals process	Interpretable AI models with detailed reasoning

Data sources: [76,77,78,79].

**Table 7:** Requirements for regulatory AI systems.

Capability Category	Specific Requirements	Implementation Complexity	Expected Timeline	Validation Requirements
Dossier Processing	Complete submission analysis with cross-referencing	High	2–3 years	95% accuracy vs. human experts
Historical Learning	Training on >50,000 regulatory submission outcomes	Very High	3–4 years	Precedent identification accuracy > 90%
Database Integration	Access to regulatory and safety databases	High	1–2 years	Real-time data synchronization
Product Specialization	Knowledge of specific therapeutic areas	High	2–3 years	Specialty-specific validation > 85%
Real-time Updates	Integration with evolving guidelines	Medium	1–2 years	Guideline compliance tracking

Data sources: [80,81,82,83].

**Table 8:** Three-stage AI development roadmap for regulatory agencies.

Development Stage	Duration	Key Activities	Deliverables	Success Metrics
Stage 1: Foundation Development	Years 1–2	Data infrastructure creation, initial AI models, pilot testing	Basic AI platform, standardized data formats, pilot results	80% document classification accuracy
Stage 2: Advanced Capabilities	Years 2–4	Machine learning enhancement, cross-module integration, international harmonization	Advanced algorithms, integration capabilities, multi-agency cooperation	90% deficiency detection accuracy
Stage 3: Comprehensive Implementation	Years 4–6	Full evaluation capability, continuous learning, quality assurance	Complete AI evaluation system, validation framework, operational deployment	95% decision accuracy compared to human experts

Timeline and metrics based on EMA’s DATUM initiative and FDA’s AI/ML Action Plan.

**Table 9:** AI ethics and security framework components.

Framework Component	Specific Requirements	Implementation Method	Validation Process
Bias Detection	Algorithmic fairness testing across demographics	Statistical parity analysis, equalized odds testing	Quarterly bias audits with third-party validation
Explainability	Clear reasoning for all AI recommendations	Interpretable AI models, decision tree visualization	Legal review of explanation adequacy
Data Security	Comprehensive cybersecurity protection	Multi-layered security architecture, encryption	Continuous monitoring, penetration testing
Accountability	Clear responsibility chains for AI decisions	Human oversight requirements, audit trails	Regular governance reviews, appeal processes

Data sources: [104,105,106,107,108,109,110,111,112,113].

**Table 10:** Individual agency implementation framework.

Implementation Component	Approach	Timeline	Success Indicators	Resource Requirements
Policy Development	Agency-specific tailoring	6–12 months	Regulatory framework adoption	USD 500 K–1 M
AI System Development	Phased technology implementation	4–6 years	System performance metrics	USD 10–25 M
Stakeholder Engagement	Industry and public consultation	Ongoing	Acceptance and compliance rates	USD 200 K–500 K annually
Performance Monitoring	Continuous outcome assessment	Ongoing	Quality and efficiency improvements	USD 300 K–800 K annually

Data sources: [114,115,116,117,118,119].

**Table 11:** Comprehensive evaluation framework components.

Evaluation Category	Assessment Areas	Measurement Methods	Success Criteria	Current Performance Data
Scientific Evaluation	Product quality, manufacturing standards, clinical performance	Analytical testing, GMP audits, post-market surveillance	>95% quality compliance	96–98% achieved in pilot programs
Economic Evaluation	Resource utilization, market access, cost-effectiveness	Cost–benefit analysis, time-to-market measurement	30–50% efficiency improvement	40–60% achieved in early implementations
Public Health Evaluation	Population health outcomes, healthcare integration	Health outcome tracking, system integration assessment	Measurable population health improvement	25–35% improvement in treatment outcomes

Data sources: [125,126,127,128,129,130,131,132,133,134,135,136].

**Table 12:** Economic analysis of quality compromises in pharmaceutical products.

Quality Issue	Direct Costs	Indirect Costs	Long-term Impact	Total Economic Burden
Treatment Failures	Higher dosing requirements	Additional healthcare interventions	Increased disease burden	150–200% of product cost
Reduced Efficacy	Extended treatment duration	Diagnostic testing needs	Patient non-compliance	120–180% of product cost
Safety Issues	Hospitalization costs	Legal liabilities	Healthcare system burden	200–300% of product cost
Quality Variations	Batch rejections	Supply chain disruptions	Market confidence loss	100–150% of product cost

Based on WHO pharmaceutical economics studies and contemporary treatment failure cost analyses.

**Table 13:** Expected public health impact of framework implementation.

Health Outcome	Baseline (Current)	Projected Improvement	Population Affected	Economic Value
Access to Quality Medicines	60–70% population coverage	85–95% population coverage	200–500 million people	USD 15–30 billion annually
Treatment Success Rates	70–80% efficacy	90–95% efficacy	All patients using affected medicines	USD 8–20 billion in avoided healthcare costs
Safety Incident Reduction	5–10% adverse events	1–3% adverse events	Medicine users globally	USD 3–12 billion in avoided harm costs
Healthcare System Efficiency	Variable quality outcomes	Standardized quality outcomes	National healthcare systems	USD 20–40 billion in system efficiencies

Data sources: [145,146,147,148,149,150,151,152].

**Table 14:** Projected benefits of framework implementation.

Benefit Category	Quantified Impact	Timeline for Realization	Measurement Method	Validation Data
Product Quality Improvement	90–95% quality standardization	2–3 years post-implementation	Comparative quality testing	96% achieved in pilot programs
Market Access Enhancement	40–60% reduction in time-to-market	1–2 years post-implementation	Regulatory approval timelines	55% average reduction in early adopters
Regulatory Capacity-building	200–300% increase in evaluation capability	4–6 years (AI development)	Processing capacity metrics	250% increase demonstrated in Brazil
Economic Efficiency	30–50% reduction in regulatory costs	2–4 years post-implementation	Cost–benefit analysis	45% cost reduction in Rwanda implementation

Data sources: [153,154,155,156].

**Table 15:** Implementation challenges and risk mitigation strategies.

Challenge Category	Specific Risks	Mitigation Strategies	Implementation Timeline
Technology Dependence	System reliability, vendor lock-in, maintenance costs	Redundant systems, open-source components, multi-vendor approach	Ongoing
Regulatory Sovereignty	Autonomy concerns, external dependencies	Flexible implementation, national customization, voluntary adoption	1–2 years
Implementation Complexity	Coordination challenges, standardization issues, resource requirements	Phased rollout, technical assistance, international support	3–5 years
Financial Sustainability	Development costs, operational expenses, revenue models	Public–private partnerships, shared costs, fee structures	2–4 years
Data Security	Cybersecurity threats, data breaches, system vulnerabilities	Comprehensive security framework, continuous monitoring, incident response	Ongoing

Data sources: [157,158,159,160,161,162].

**Table 16:** AI system quality assurance and validation framework.

Validation Component	Assessment Method	Acceptance Criteria	Monitoring Frequency	Current Performance
Accuracy Assessment	Comparative analysis with human experts	>95% concordance	Continuous	96–98% in pilot programs
Reliability Testing	Cross-validation with multiple agencies	>90% consistency	Quarterly	94% consistency achieved
Bias Detection	Algorithm fairness assessment	No systematic bias detected	Semi-annually	No significant bias detected
Security Evaluation	Cybersecurity and data protection audit	Full compliance with standards	Annually	100% compliance maintained

Data sources: [163,164].

**Table 17:** Recommended implementation timeline and milestones.

Implementation Stage	Duration	Key Activities	Success Milestones	Resource Requirements
Preparation and Planning	6–12 months	Stakeholder engagement, policy development, resource mobilization	Framework adoption, funding secured	USD 2–5 million
Foundation Development	12–24 months	AI system basic development, pilot testing, initial training	Basic system operational	USD 15–30 million
Advanced Implementation	24–36 months	Full system deployment, staff training, process integration	System fully operational	USD 20–40 million
Optimization and Expansion	36–48 months	Performance optimization, international cooperation, knowledge sharing	Regional adoption, efficiency gains	USD 8–20 million

Data sources: Contemporary implementation cost analyses and timeline studies.

**Table 18:** Framework success metrics and performance indicators.

Metric Category	Key Performance Indicators	Target Values	Measurement Frequency	Current Achievement
Quality Outcomes	Pharmaceutical compliance rates, safety signal detection, therapeutic success rates	>95% compliance, <48 h signal detection, >90% efficacy	Monthly	96% compliance in pilots
Regulatory Efficiency	Review timeline reduction, cost savings, stakeholder satisfaction	50% faster reviews, 40% cost reduction, >80% satisfaction	Quarterly	55% timeline reduction achieved
Public Health Impact	Population access improvement, health outcome enhancement, economic benefits	30% access increase, 20% outcome improvement, USD 5B annual savings	Annually	35% access increase in early adopters
System Performance	AI accuracy, decision consistency, capacity utilization	>95% accuracy, >90% consistency, >80% utilization	Real-time	96% accuracy demonstrated

Data sources: [64,165,166,167,168].

## Data Availability

No new data were created or analyzed in this study. Data sharing is not applicable.
